# TGFBI promoter hypermethylation correlating with paclitaxel chemoresistance in ovarian cancer

**DOI:** 10.1186/1756-9966-31-6

**Published:** 2012-01-16

**Authors:** Ning Wang, Hui Zhang, Qin Yao, Yankui Wang, Shuzhen Dai, Xingsheng Yang

**Affiliations:** 1 Department of Obstetrics and Gynecology, Qilu Hospital, Shandong University, 107 Wenhuaxi Road, Jinan 250012, P.R. China; 2 Department of Obstetrics and Gynecology, Affiliated Hospital of Medical College, Qingdao University, 16 JiangSu Road, Qingdao 266003, P.R. China

**Keywords:** Ovarian cancer, transforming growth factor-beta-inducible gene-h3, methylation, chemoresistance, paclitaxel

## Abstract

The purpose of this study is to determine the methylation status of Transforming growth factor-beta-inducible gene-h3 (TGFBI) and its correlation with paclitaxel chemoresistance in ovarian cancer. The methylation status of TGFBI was examined in ovarian cancer and control groups by methylation-specific PCR (MSP) and bisulfite sequencing PCR (BSP). The TGFBI expression and cell viability were compared by Quantitative Real-Time PCR, Western Blotting and MTT assay before and after demethylating agent 5-aza-2'-deoxycytidine (5-aza-dc) treatment in 6 cell lines (SKOV3, SKOV3/TR, SKOV3/DDP, A2780, 2780/TR, OVCAR8). In our results, TGFBI methylation was detected in 29/40 (72.5%) of ovarian cancer and 1/10 (10%) of benign ovarian tumors. No methylation was detected in normal ovarian tissues (*P *< 0.001). No statistical correlation between RUNX3 methylation and clinicopathological characteristics was observed. A significant correlation between TGFBI methylation and loss of TGFBI mRNA expression was found (*P *< 0.001). The methylation level of TGFBI was significantly higher in paclitaxel resistant cell lines (SKOV3/TR and 2780/TR) than that in the sensitive pairs (*P *< 0.001). After 5-aza-dc treatment, the relative expression of TGFBI mRNA and protein increased significantly in SKOV3/TR and A2780/TR cells. However, no statistical differences of relative TGFBI mRNA expression and protein were found in other cells (all *P *> 0.05), which showed that re-expression of TGFBI could reverse paclitaxel chemoresistance. Our results show that TGFBI is frequently methylated and associated with paclitaxel-resistance in ovarian cancer. TGFBI might be a potential therapeutic target for the enhancement of responses to chemotherapy in ovarian cancer patients.

## Introduction

Epithelial ovarian cancer is the most lethal gynecologic malignancy, with 21 990 estimated new cases and 15 460 deaths in the USA in 2011 [1]. Reasons for this high lethality include the advanced stage at which patients are diagnosed and the inherent aggressive biology of this cancer.

Maximal surgical cytoreduction followed by systemic chemotherapy with carboplatin and paclitaxel is the current standard treatment modality for advanced ovarian cancer [2]. A key feature of ovarian cancer is its sensitivity to chemotherapeutic drugs such as paclitaxel, a prototype taxane, stabilizes microtubule polymers leading to mitotic arrest and apoptosis [3]. Unfortunately, ovarian cancer cells, with their unstable genomes [4], are initially sensitive to these drugs, but long term utilization may result in the chemoresistance [5].

Epigenetic alterations play an important role in the initiation and progression of cancer [6-8]. Hypermethylation of CpG rich islands in promoter regions of genes has been characterized as a common epigenetic alteration for the silencing or inactivation of tumor suppressor genes and transcriptional repression in human malignancies [9,10], including ovarian cancer [11-13]. In recent years, emerging evidence has also linked epigenetic changes to the development of drug resistance [14,15].

Transforming growth factor-beta-inducible gene-h3 (TGFBI) is a secreted protein first identified in a human lung adenocarcinoma cell line treated with transforming growth factor-β [16]. It has been shown to possess tumor suppressor function in vitro studies [17,18], and to be correlated with specific sensitization to paclitaxel by inducing stabilization of microtubules via integrin-mediated signaling pathways [19]. Recently, promoter hypermethylation of TGFBI was found in lung [20,21] and prostate cancer [20]. However, the role of TGFBI methylation in paclitaxel chemoresistance in ovarian cancer is unknown. Therefore, a better understanding of this epigenetic mechanism of TGFBI in ovarian cancer could facilitate the generation of new drugs that re-sensitize tumor cells to paclitaxel [4].

In this study, we examined the methylation status and expression of TGFBI in epithelial ovarian cancer tissues, paclitaxel-sensitive and -resistant ovarian cancer cell lines in order to determine whether the methylation of TGFBI is asscociated with paclitaxel chemoresistance.

## Materials and methods

### Ovarian cancer tissue samples and cell lines

From April 2008 to April 2009, 40 primary epithelial ovarian cancer(, 10 benign tumor and 10 normal ovarian tissues) were collected at the Department of Obstetrics and Gynecology, The Affiliated Hospital of Medical College, Qingdao University, China. The mean age of the patients was 43 years (range 21-77 years). The ovarian cancer patients have different histological types: serous papillary carcinoma (n = 20), mucinous carcinoma (n = 13), endometrioid carcinoma (n = 7). Six patients were in stage I, ten patients were in stage II, twenty-four patients were in stage III. Twenty-two patients had metastasis to pelvic lymph nodes. Eleven tumors were well-moderately differentiated, and 29 tumors were poorly differentiated. Ten benign tumor and 10 normal ovarian tissues were collected as control. All samples were obtained prior to chemotherapy or radiation therapy, which were placed in liquid nitrogen immediately after resection and stored at -80°C until use. The malignant and normal diagnosis was performed by pathologists. The study was performed after approval by our institute Medical Ethics Committee.

Human SKOV3, A2780 and OVCAR8 ovarian cancer cell lines were obtained from the bioengineering centre of The Affiliated Hospital of Medical College, Qingdao University, China. The chemoresistant cell lines (SKOV3/DDP, SKOV3/TR, and A2780/TR) were purchased from the China Center for Type Culture Collection (Wuhan, China). These cells were maintained in DMEM with 10% fetal bovine serum and 100 U/ml penicillin/streptomycin at 37°C. SKOV3/TR and A2780/TR were cultured in RPMI-1640 medium containing 0.3 μmol/L paclitaxel to maintain the drugresistant phenotype.

Cells were grown to 70% confluence and treated with 10 μmol/L of demethylating agent (5-aza-2'-deoxycytidine, 5-aza-dc) (Sigma-Aldrich, St. Louis, MO, USA) for 3 days [22]. After the treatment, cells were harvested and extracted for DNA, RNA and protein.

### Nucleic acid isolation

The EZNA Tissue DNA Kit (Omega Corp, USA) was used to extract high purity DNA from different ovarian tissues and ovarian cancer cell lines. Total DNA content was quantified by UV absorbance value measured at A260 and A280, and diluted to a concentration of 1 μg/100 μl.

### Methylation-specific PCR (MSP) and bisulfite sequencing PCR (BSP)

DNA from tissue samples and cell lines were subjected to bisulfite treatment using CpGgenome DNA Modification Kit (Chemicon, USA). Sequences, Tm, and product length of each primer used for MSP and BSP analysis are summarized in Table 1 The band expanded with methylation-specific PCR primers corresponding to the DNA methylation in the promoter region was marked as "M". The band expanded with non-methylation-specific primers was marked as "U".

**Table 1:** Sequences of Primers for MSP and RT-PCR

Gene	Primer	Sequences	Temp (°C)	Length(bp)
TGFBI	M-F	5'-gAAAATTGAGTACGGGTATAGTGC-3'	58	162
	M-R	5'-CCAAATTAAATAAACTACGAACGAA-3'		
	U-F	5'-AAAATTGAGTATGGGTATAGTGTGG-3'	56	161
	U-R	5'-CCAAATTAAATAAACTACAAACAAA-3'		
	B-F	5'-TGGAAGTAGTTATAGGAGGTTTAAGG-3'	55	402
	B-R	5'-CCCAAAACCAAAACCAAAAC-3'		
	qRT-F	5'-GGCTGCAGAGTCTGATGTGT-3'	55	81
	qRT-F	5'-CGCTCACTTCCAGAGAGATG-3'		
GAPDH	qRT-F	5'-GGACCTGACCTGCCGTCTAG-3'	55	99
	qRT-R	5'-TAGCCCAGGATGCCCTTGAG-3'		

M: methylated primers [23], U: unmethylated primers [23], B: primers of BSP primers (designed using MethPrimer), qRT, primers of Quantitative Real-Time PCR.

### Quantitative real-time PCR (qRT-PCR)

Total RNA was extracted from cells with Trizol reagent (Invitrogen, San Diego, CA, USA), and it was reverse transcribed using miScript Reverse Transcription Kit (Qiagen, Hilden, Germany). The primers for mRNA are listed in Table 1. The quantification was performed with QuantiTect Probe RT-PCR (Qiagen, Hilden, Germany). The comparative threshold cycle method was used to determine gene relative expression.

### Western blotting

Cells were washed twice with ice-cold phosphate-buffered saline and lysed using a modified RIPA buffer supplemented with 1 mM PMSF. The protein concentration was detected using BCA protein assay (Pierce, Rockford, IL, USA). Proteins were loaded onto 10% and 5% SDS-PAGE and electrophoretically transferred to a PVDF membrane (Millipore, Bedford, MA, USA). After blocking with 5% non-fat milk in PBS-Tween 20 for 2 h at room temperature, the membranes were incubated with anti-human monoclonal β-actin and anti-human TGFBI primary antibody overnight at 4°C. Horseradish peroxidase-conjugated secondary antibody was added for 2 h at room temperature. The Detection was performed by chemiluminescence.

### MTT assay

MTT Cell Proliferation Assay (Biosharp, USA) was used to measure cell viability. Before and after treated with 5-aza-dc, 1 × 10^4 ^cells/well were seeded in 96-well plates containing complete medium and incubated for 24 h. Then cells were exposed to serial dilutions of paclitaxel in a total volume of 200 μL in four replicate wells. After 48 hours, plates were added 20 μl of MTT reagent and incubated for 4 h, and then formazane crystals formed were dissolved in 150 μl of dimethyl sulfoxide (Wako, Tokyo, Japan). The optical density was measured at 490 nm on a microplate reader. The half maximal inhibitory concentration (IC50) value was assessed by different concentrations of paclitaxel (0.01, 0.1 and 1 μM).

### Statistical analyses

All statistical analyses were performed using SPSS 15.0. Fisher's exact test or and *χ^2 ^*test were used to compare TGFBI methylation status among cases and between various clinicopathologic variables. Pearson correlation analysis was used to evaluate the relationship between TGFBI methylation status and mRNA expression. The differences of TGFBI mRNA and protein expression before and after 5-aza-dc treatment were analyzed by the Paired-Samples *t *test. *P *< 0.05 was considered statistically significant.

## Results

### Frequency of TGFBI methylation in ovarian cancer tissues

**Figure 1:**
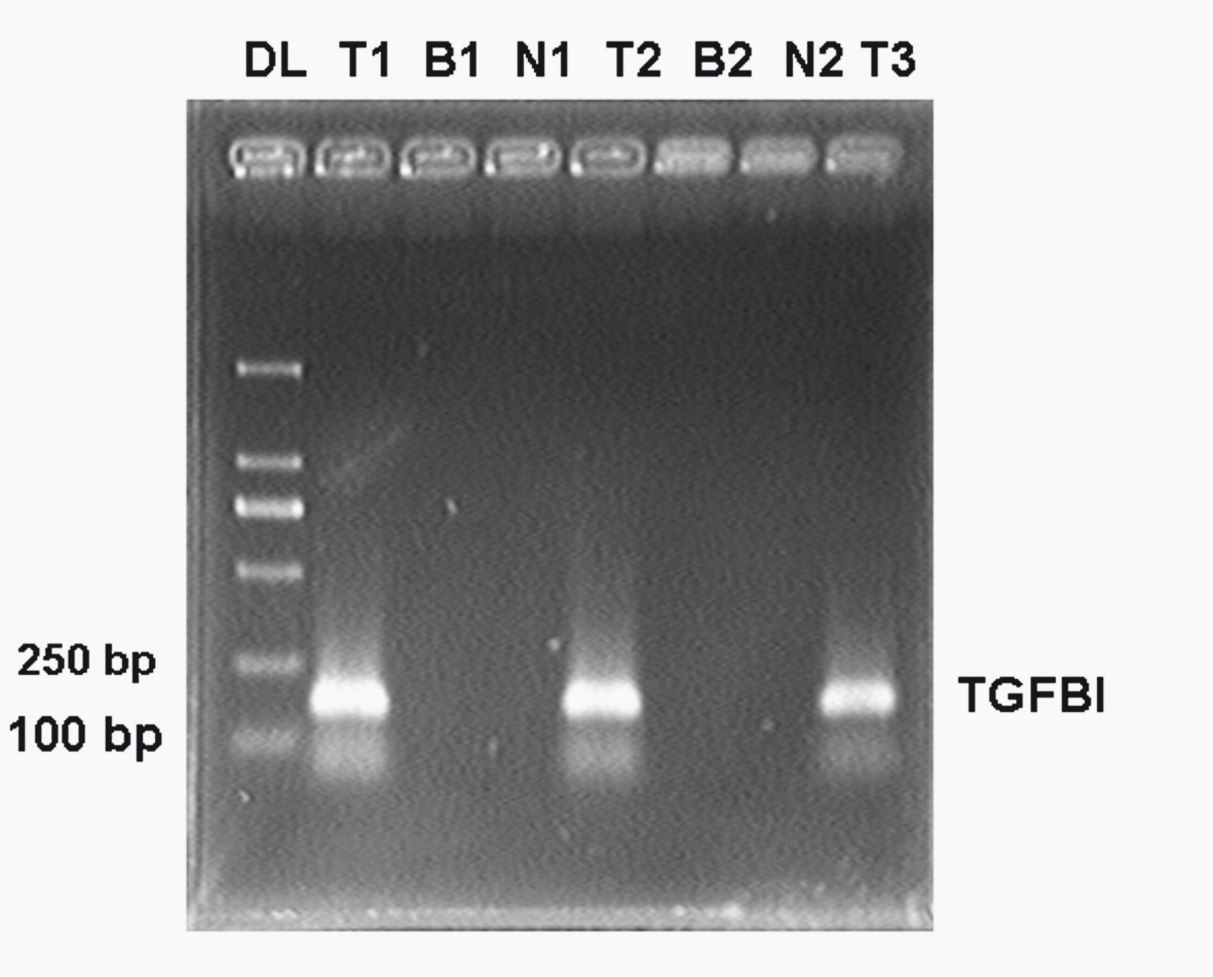
**Methylation status of TGFBI in ovarian cancer, benign ovarian cancer and normal ovarian cancer tissues**. Three carcinomas had completely methylated TGFBI genes, while 2 benign and 2 normal cases showed no methylation. DL: Marker DL2000; T1, T2, T3: ovarian cancer tissues; B1, B2: benign ovarian tissues; N1, N2: normal ovarian tissues.

We determined the frequency of TGFBI methylation in 40 primary ovarian cancer samples, 10 benign ovarian tumors and 10 normal ovarian tissues by MSP (Figure 1). TGFBI in all of the normal ovarian tissues was completely methylation free, which showed success of DNA modification. The overall frequency of methylation in benign ovarian tumors was 10.0% (1/10). For ovarian cancer tissues, 72.5% (29/40) of methylation was observed. The data demonstrated that the difference of TGFBI methylation frequency among ovarian cancers, benign ovarian tumors and normal ovarian tissues was statistically significant (*P *< 0.001).

The methylation status of the ovarian cancers was compared with clinicopathological characteristics from these patients including age, histological type, tumor stage, histological grade and lymphatic metastasis. No significant correlation between TGFBI methylation and any of these parameters was observed for the ovarian cancer patients (Table 2).

**Table 2:** Association of TGFBI methylation and clinicopathologic variables in 40 ovarian cancer patients

Clinicopathologic characteristics	Number (n)	Methylation (%)	*P *value
Age at diagnosis			
< 50 years	14	9 (64.3)	0.3932
≥50 years	26	20 (76.9)	
Histological type			
Serous adenocarcinoma	20	16 (80.0)	0.4814
Mucinous adenocarcinoma	13	9 (69.2)	
Endometrioid adenocarcinoma	7	4 (57.1)	
Tumor stage			
I	6	2 (33.3)	0.0661
II	10	8 (80.0)	
III	24	19 (79.2)	
Histological grade			
G1	4	2 (40.0)	0.5532
G2	7	5 (71.4)	
G3	29	22 (75.9)	
Lymphatic metastasis			
No	18	13 (72.2)	0.9716
Yes	22	16 (72.7)	

### Expression of TGFBI mRNA in ovarian cancer tissues

To examine whether TGFBI methylation results in the suppression of TGFBI expression, we examined TGFBI mRNA expression by qRT-PCR in 40 ovarian cancer tissues and 10 normal ovarian tissues. TGFBI mRNA expression was detected in all the normal ovarian tissues (10/10) and in most of the unmethylated ovarian cancer tissues (10/11). In contrast, TGFBI expression was not detected in the TGFBI-methylated ovarian cancer tissues (27/29), except for 2 tissues. We compared the TGFBI mRNA expression results of these ovarian cancer tissues with the TGFBI methylation data and found a significant correlation between TGFBI methylation and loss of TGFBI mRNA expression (*P *< 0.001). These results suggest that the inactivation of TGFBI expression is closely correlated with gene methylation in ovarian cancer tissues.

### Demethylation and re-expression of TGFBI after treating with 5-aza-dc in ovarian cancer lines

**Figure 2:**
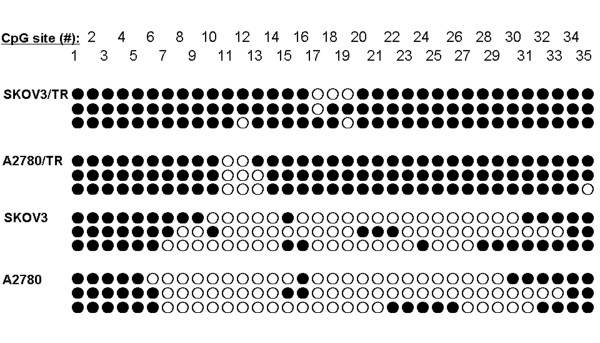
**Bisulfite sequencing of SKOV3, SKOV3/TR, A2780 and A2780/TR**. Paclitaxel-resistant cell lines (SKOV3/TR and A2780/TR) showed almost complete CpG methylation (91.4% and 97.1% of total CpG sites, respectively), the sensitive cell lines SKOV3 and A2780 showed partial methylation of CpG islands (42.9% and 35.24% of total CpG sites, respectively).

We detected the methylation status of TGFBI promoter region in 4 ovarian cell lines by MSP and BSP before and after treating with 5-aza-dc. Before treatment, there was partial TGFBI methylation detected in SKOV3 and A2780 cells (42.9% and 35.2% of total CpG sites, respectively). In contrast, almost complete hypermethylation were found in SKOV3/TR (94.3% of total CpG sites) and A2780/TR (91.4% of total CpG sites) cells (Figure 2).

**Figure 3:**
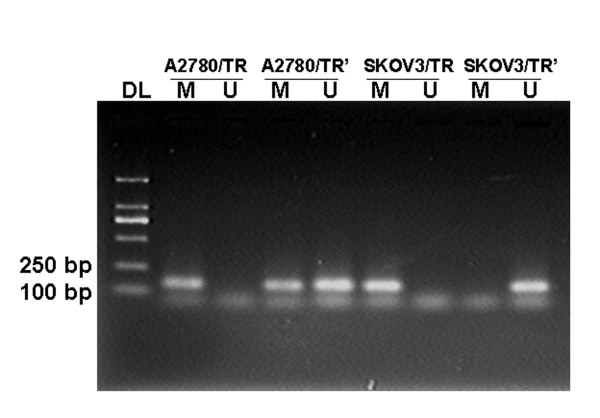
**MSP analysis of TGFBI in paplitaxel resistant cell lines after demethylation by 5-aza-dc**. After 5-aza-dc treatment, a "U" band appeared while the "M" band did not disappear in A2780/TR cell line. In another cell line SKOV3/TR, the "M" band disappeared and only a "U" band was left. DL: Marker DL2000; SKOV3/TR, A2780/TR: before treatment; SKOV3/TR', A2780/TR': after treatment; U: unmethylation, M: methylation.

After 5-aza-dc treatment, a "U" band appeared while the "M" band did not disappear in A2780/TR cell line, which demonstrated that the methylation was partially reversed. In another cell line SKOV3/TR, the "M" band disappeared and only a "U" band was left, indicating that the methylation had been completely reversed (Figure 3).

**Figure 4:**
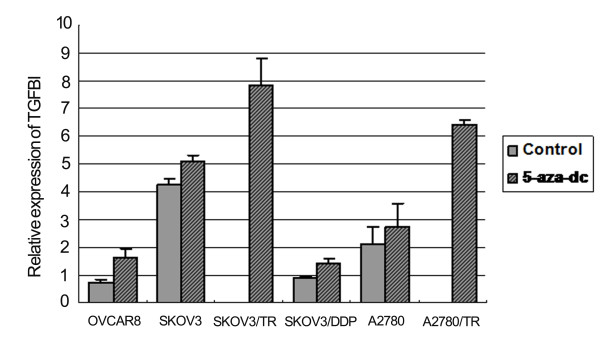
**Quantitative real-time RT-PCR analysis of TGFBI expression in ovarian cancer cells**. It showed that the relative expression of TGFBI mRNA increased significantly after treating with 5-aza-dc in SKOV3/TR and A2780/TR cells. However, no statistical differences of relative TGFBI mRNA expression were found after 5-aza-dc administration in other cell lines.

The expression of TGFBI mRNA was examined in all the 6 ovarian cancer cell lines by qRT-PCR before and after treating with 5-aza-dc (Figure 4). Our data showed that the relative expression of TGFBI mRNA increased significantly after treating with 5-aza-dc in SKOV3/TR (7.8 ± 0.9 vs. 0, *P *< 0.001) and A2780/TR (6.4 ± 0.2 vs.0, *P *< 0.001) cells. However, no statistical differences of relative TGFBI mRNA expression were found after 5-aza-dc administration in OVCAR8 (1.6 ± 0.3 vs. 0.8 ± 0.1, *P >*0.05), SKOV3(5.1 ± 0.2 vs. 4.2 ± 0.2, *P >*0.05), SKOV3/DDP (1.4 ± 0.9 vs. 0.9 ± 0.2, *P >*0.05) and A2780 cells 2.7 ± 0.9 vs. 2.1 ± 0.7, *P >*0.05).

**Figure 5:**
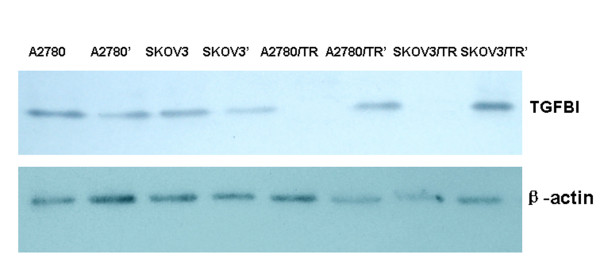
**The TGFBIp expression before and after treatment of 5-aza-dc by Western blotting**. Expression of TGFBIp in SKOV3/TR and A2780/TR cell lines was sharply up-regulated after treatment of 5-aza-dc. A2780, SKOV3, A2780/TR, SKOV3/TR: before treatment; A2780', SKOV3', A2780/TR', SKOV3/TR': after 5-aza-dc treatment.

In addition, we examined TGFBI protein (TGFBIp) expression in all the cell lines by Western blotting (Figure 5). The data showed that the expression of TGFBIp in SKOV3/TR and A2780/TR cell lines was statistically up-regulated after 5-aza-dc administration (*P *< 0.01 and *P *< 0.01, respectively). By contrast, no significant differences were found in other cell lines (all *P *> 0.05), which was coincident with the results of qRT-PCR.

### MTT assay

**Figure 6:**
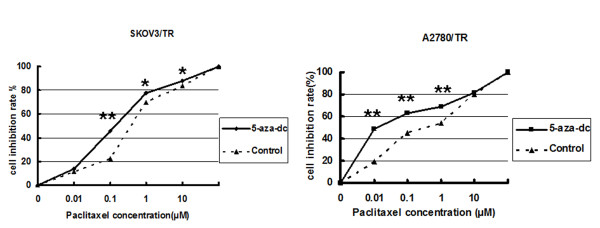
**Demethylation of TGFBI restores the sensitivity of paclitaxel-resistant ovarian cells**. The inhibition rates in paclitaxel-resistant cells with 5-aza-dc treatment were increased significantly than control ones (* *P *< 0.05; ** *P *< 0.01).

Further, we studied the effect of cell proliferation of 5-aza-dc on paclitaxel-resistant cell lines by MTT viability assay. Our results showed that the rate of cell inhibition was significantly increased in SKOV3/TR and A2780/TR than that in control groups at several paclitaxel concentrations of 0.01, 0.1 and 1 μM (*P *< 0.05) (Figure 6). The IC50 of SKOV3/TR obviously decreased after 5-aza-dc administration (0.19 ± 0.01 μM vs. 0.42 ± 0.02 μM, *P *= 0.001), which was similar with the results of A2780/TR (0.012 ± 0.0001 μM vs. 0.33 ± 0.011 μM; *P *= 0.001).

## Discussion

In this study, we first detected the methylation status of the 5' CpG island of TGFBI in different ovarian tissues using MSP and BSP in order to determine whether TGFBI inactivation by DNA methylation is characteristic of human ovarian cancer. After repeated experiments, our results showed that the TGFBI is frequently methylated in ovarian cancer. Its methylation can be used as a novel epigenetic biomarker for ovarian cancer detection.

We further measured TGFBI mRNA and protein levels by RT-PCR and IHC in ovarian cancer tissues. Then we compared the TGFBI expression results with the TGFBI methylation data and found a significant inverse correlation between TGFBI methylation and TGFBI expression, which confirmed the important role of promoter methylation in regulating TGFBI expression. However, because 1 ovarian cancer tissue lacking TGFBI mRNA expression was not methylated, we presume that mechanisms of inactivating the gene other than methylation must exist.

Recently, Shah et al. [20] reported that TGFBI methylation was associated with tumor recurrence and metastasis, suggesting that TGFBI is required to suppress the aggressiveness of prostate and lung cancer. In our study, the methylation rate of carcinomas with poor differentiation was higher than those with well differentiation. Meanwhile, higher methylation rate was also found in late stage patients with ovarian cancers, though no significant correlation was found between TGFBI methylation status and clinicopathological characteristics, which was in accordance with the results of Kang et al [23]. Our results showed that there were different patterns of mythylation according to the histology and the tumor grade, and revealed that hypermethylation of TGFBI in ovarian cancer might be associated with unfavourable prognosis. Further studies with large sample size and long-term follow-up are required to confirm the hypothesis.

Chemoresistance is the major cause of treatment failure for ovarian cancer. It is reported that DNA methylation may act as a potential cause of chemotherapy drug resistance [24-26]. In a recently study by Li et al. [27], cisplatin-sensitive and -resistant ovarian cancer cells were analyzed by methylation and mRNA expression microarray. Their results revealed that DNA hypermethylation may contribute to the onset of the chemoresistance in ovarian cancer.

In our study on cell lines, almost complete methylation pattern of the TGFBI promoter in 2 paclitaxel-resistant cell lines (SKOV3/TR and A2780/TR) was observed, with a complete loss or low level of TGFBI expression in these cell lines. In contrast, only sparsely methylated or unmethylated CpG sites were identified in cell lines with a rich level of TGFBI expression, including SKOV3, A2780, OVCAR8, and SKOV3/DDP ovarian cancer cell lines. Our results identified strong relation between TGFBI expression and response to chemotherapy. To our knowledge, this is the first evidence of TGFBI hypermethylation as a mechanism of paclitaxel chemoresistance in ovarian cancer.

Further, our results were confirmed by using DNA methylation inhibitors. The relative expression of TGFBI mRNA and protein increased significantly after treating with 5-aza-dc in palitaxel-resistant cells. However, no statistical differences of TGFBI expression were found after 5-aza-dc administration in other 4 cell lines. In addition, MTT assay showed that the rate of cell inhibition was significantly increased in SKOV3/TR and A2780/TR after 5-aza-dc treatment, which suggested that chemotherapy sensitivity to paclitaxel was enhanced and chemoresistance was reversed.

In conclusion, our study indicated that promoter hypermethylation of TGFBI is a frequent event in ovarian cancer. TGFBI methylation was associated with paclitaxel chemoresistance, and it can be used as a potential epigenetic biomarker and therapeutic target of paclitaxel resistance in ovarian cancer.

## Competing interests

The authors declare that they have no competing interests.

## Authors' contributions

NW and XSY designed and coordinated the study, carried out data interpretation, and drafted the manuscript; HZ participated in the conception and design of the study, and participated in drafting of manuscript; QY participated in the design of the study and performed the statistical analysis; SZD and YKW conceived of the study, and participated in its design and coordination and helped to draft the manuscript. All authors read and approved the final manuscript.
